# 3D Dental Subsurface Imaging Using Enhanced Truncated Correlation-Photothermal Coherence Tomography

**DOI:** 10.1038/s41598-019-53170-w

**Published:** 2019-11-14

**Authors:** Sohrab Roointan, Pantea Tavakolian, Koneswaran S. Sivagurunathan, Marie Floryan, Andreas Mandelis, Stephen H. Abrams

**Affiliations:** 1 Center for Advanced Diffusion-Wave and Photoacoustic Technologies (CADIPT), Department of Mechanical and Industrial Engineering (MIE)and Institute of Biomaterials and Biomedical Engineering (IBBME), University of Toronto, Toronto, Ontario M5S3G8 Canada; 2 Quantum Dental Technologies, 748 Briar Hill Avenue, Toronto, ON M6B 1L3 Canada

**Keywords:** Minimal intervention dentistry, Three-dimensional imaging, Biomedical engineering, Imaging and sensing

## Abstract

Development of accurate and sensitive dental imaging technologies is a top priority in the pursuit of high-quality dental care. However, while early dental caries detection and routine monitoring of treatment progress are crucial for effective long-term results, current radiographic technologies fall short of this objective due to low sensitivity for small lesions and use of ionizing radiation which is unsuitable for frequent monitoring. Here we demonstrate the first application of enhanced Truncated Correlation-Photothermal Coherence Tomography (eTC-PCT) to dental imaging. eTC-PCT is non-invasive and non-ionizing, operates well below the maximum permissible exposure (MPE) limit, and features 3D subsurface imaging capability with operator controlled axial resolution. We explore the potential of this method for dental applications and demonstrate its capability for depth-resolved tomographic 3D reconstructions of the details and subsurface extent of a variety of dental defects. To this end, in this proof-of-concept study, dental eTC-PCT imaging results, and its sensitivity to dental caries, are discussed in comparison with visual examination, x-rays and micro-CT imaging.

## Introduction

Dental complications are among the most prevalent and costly health problems in human population, with dental caries being the most common oral disease in the United States^1,2^. Currently, dental diagnosis is primarily done through visual inspection by a dentist, and confirmed with the aid of dental x-rays. This routine, however, has well known limitations. Often, visual inspection proves insufficient for detecting early caries and the extent of more advanced decay conditions^3^. Additionally, while commonly used imaging diagnostic technologies, mainly X-rays, enable visualization of general tooth structure and its features, they are unable to reliably detect dental caries^3^ (especially at its onset) and are unsuitable for routine monitoring due to harmful radiation. X-rays are mostly limited to detecting lesions in the interproximal or contact regions, once the lesions are at least halfway through the enamel shell. They are frequently incapable of locating lesions found on occlusal surfaces^4–6^, and those beneath tooth surfaces perpendicular to the x-ray beam direction (i.e. buccal or lingual surfaces in clinical settings), since the carious region is often sandwiched between healthy layers^7^. Furthermore, regardless of the caries stage at which a dentist’s intervention is sought by the patient, routine and accurate post-treatment monitoring is key for properly maintaining and improving the patient’s dental health.

Nevertheless, the aforementioned shortcomings in the common diagnostic procedures often lead to missed opportunities for re-mineralization of early caries lesions^8^, and the recurrence of caries after invasive intervention due to a lack of frequent, accurate monitoring. As such, there exists the need for a suitable dental imaging technology to combine structural visualization capability, high sensitivity and safety for routine exposure of the patient, to promptly address these limitations.

Over the past three decades, efforts have been made to develop non-ionizing optical and thermal techniques as alternative methods for caries detection and dental imaging^9–17^. Of these methods, those currently available for clinical use mainly employ transillumination, laser fluorescence or thermophotonics. However, while these approaches have had promising results, they each have certain disadvantages as well. Near-infrared transillumination-based imaging methods provide highly detailed images and have the potential to greatly reduce the use of ionizing x-ray; however, similar to x-rays, they rely on the dentist’s subjective visual assessment of grey shadows in 2D images^16^. Fluorescence-based methods such as quantitative light-induced fluorescence (QLF), which features robust caries detection performance^18^, are limited in penetration depth due to their purely optical nature which is set back by the rapid absorption and scattering of incident photons. As well, a number of other methods based on laser fluorescence, further exhibit the major limitation of only assessing bacterial porphyrin presence, and not the actual demineralization in tooth crystal structure^19^, thus resulting in many false positives. Additionally, none of these methods are capable of providing depth profilometric information for the lesion^20,21^. The thermophotonics-based PTR-LUM (“Photothermal Radiometry and Modulated Luminescence”) point-measurement technology^14,15^, assesses the crystal structure of the tooth, and has proven to be reliable for clinical early caries detection and characterization^21,22^. However, this technology is also not a depth profilometric method because it operates with laser beam illumination at a fixed modulation frequency.

While not yet adopted for clinical use, Optical Coherence Tomography (OCT) is an emerging dental imaging approach, which can detect enamel demineralization with high lateral/axial resolution, and is capable of depth profilometry. To our knowledge, all applications of OCT to dental imaging have been in either 1300 nm range (the majority of studies) or 850 nm range. As well, swept-source cross polarized (CP)-OCT at 1310 nm has recently been employed to generate 3D tomographic images of teeth with occlusal lesions^23^. However, a recent comparison of 1310 nm (CP)-OCT and 808 nm thermophonic lock-in (TPLI) imaging (in the 8–14 μm spectral range) has shown superior sensitivity and detection threshold for the thermophotonics-based method in early caries detection^24^. Furthermore, OCT penetration depth, particularly in the λ < 1,000 nm range (~1.3 mm at 840 nm^25^), is limited by the purely optical nature of the method, which solely depends on photon penetration.

Building on the high sensitivity of 808 nm TPLI to dental caries, we present here the first application of the recently developed enhanced Truncated Correlation-Photothermal Coherence Tomography (eTC-PCT)^26^ thermophotonics-based 3D imaging technology to dental imaging. This technology is an evolution of the original TC-PCT method^27^, which has been shown to be capable of monitoring bone mineral loss^28,29^. The eTC-PCT modality has enabled significantly higher depth-profiling capabilities with industrial materials^26,30,31^ compared to the original TC-PCT and to today’s state-of-the-art dynamic photothermal imaging technologies, namely lock-in thermography and photothermal radar. Notably, the capability of eTC-PCT for biomedical imaging, and its advantage over TC-PCT in this application, has been recently demonstrated for *in-vivo* tumor detection in a mouse^32^. The eTC-PCT system output consists of consecutive thermal images (slices), each corresponding to a different depth/signal delay. Compilation of slices with subsurface depth as a parameter results in 3D reconstruction. To increase the signal-to-noise ratio (SNR) of photothermal images, eTC-PCT uses a chirped pulse excitation with fixed pulse width, performs pulse-compression, and uses a time- evolving filter controlled by pulse delay and time slicing width^26^. Compared to the original TC-PCT, the enhanced version employs a highly optimized algorithm for 3D reconstruction. The new algorithm adds a time gating filter to localize the distribution of energy, whereas the conventional TC-PCT algorithm truncates the excitation signal to obtain the image slices. While the conventional TC-PCT output images (slices) were limited to the energy localized into a very narrow slice width smaller than the excitation pulse-width, the modified reconstruction algorithm proposed in eTC-PCT removes this limitation. This feature enables the localization of a larger amount of energy in each slice, resulting in higher signal quality and thus higher dynamic range compared to TC-PCT. Through these optimizations, eTC-PCT has been shown to achieve a penetration depth 2.6 times that of TC-PCT in steel^26^.

In this study, eTC-PCT spatial reconstruction features and detection capabilities are compared to X-rays in various tooth conditions (structural damage due to advanced decay, early enamel caries and amalgam restoration). We have optimized the eTC-PCT image acquisition and laser parameters for dental applications and assessed its effectiveness, making it the first thermophotonic imaging technique capable of 3D tomographic subsurface dental characterization. To this end, we have investigated the data from three different eTC-PCT output channels; amplitude, phase and zero-phase delay time^26^. As a result of the foregoing adaptation of eTC-PCT to dental imaging, we have generated the first 3D reconstructions of a highly turbid medium (teeth) from the eTC-PCT zero-phase delay time output data, which proves to be a critical indicator in measuring depth profiles of defective regions in teeth.

The high contrast/high sensitivity and subsurface depth profilometry capability of dental eTC-PCT in the 808 nm range, coupled with its lack of ionizing radiation, absence of moving parts, and maximum-permissible exposure (MPE) compatibility^33^, makes it highly suited to dental imaging. This study is the first step towards further developing this technology as a potential early dental caries detection and characterization modality, and a wide-utility safe tool for tooth integrity inspection of regions beyond the capability of currently utilized dental imaging technologies.

## Results

### eTC-PCT overview

**Figure 1:**
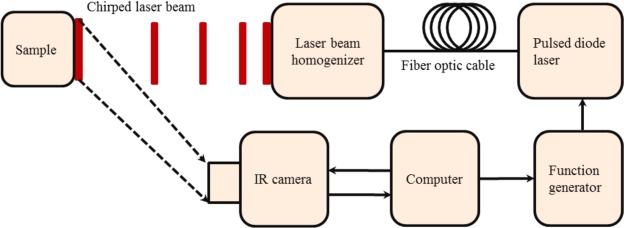
eTC-PCT experimental setup schematic.

**Figure 2:**
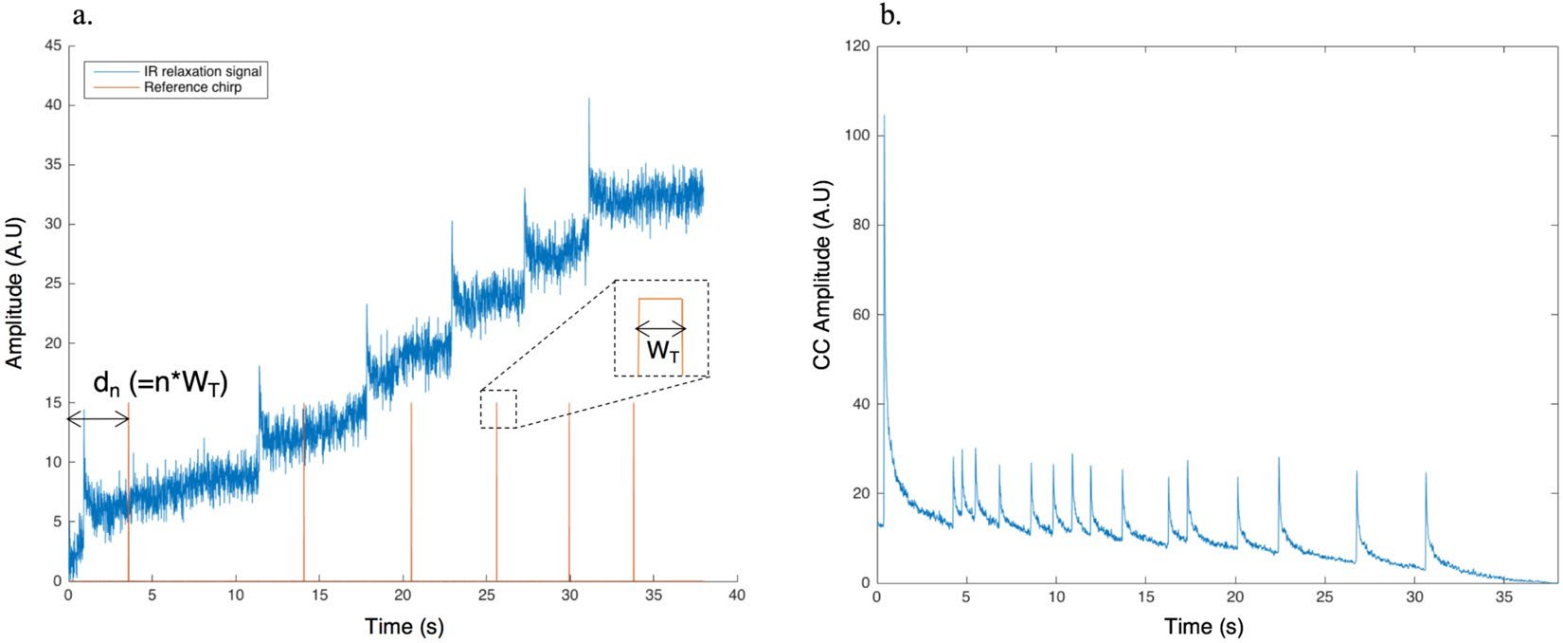
eTC-PCT slice calculation. (**a**) Typical raw thermal relaxation signal recorded by the IR camera for one pixel (in blue), and the corresponding synthesized reference chirp after delay unit d_n_ (in orange). Slice width W_T_ is selected by the operator and the delay unit d_n_ is incremented as multiples “n” of W_T_, where “n” is the slice number. Cross correlation is calculated at each d_n_. (**b**) Typical cross-correlation result of the synthesized reference chirp signal and the IR thermal relaxation signal for one pixel at d = 0 (i.e. the initial slice with n = 0). Note the very narrow FWHM of the CC, the key feature of high axial resolution of eTC-PCT despite the diffusive nature of the thermal wave.

The eTC-PCT imaging set-up is shown in Fig. 1. The sample is placed on the imaging platform at the focal point of a mid-IR camera. The camera records the photothermal evolution of the sample following exposure to linear frequency modulated (LFM) chirp pulse laser irradiation, with an incident beam diameter of ~24 mm and excitation pulse width between 20 ms to 80 ms, depending on the desired detection depth. The corresponding energy density per pulse used in this study ranged from ~0.26 J/cm^2^ to a maximum of ~1.03 J/cm^2^ for the 80-ms pulse, which resulted in a maximum temperature increase of 0.8 °C in the tooth pulp after the full chirp. The energy density values are in compliance with MPE requirements and, in particular, are less than 50% MPE for shorter pulse widths. The pulse duration is considerably shorter than the thermal relaxation time, leading to a thermally confined absorber localization process. Briefly, in eTC-PCT, a reference chirp signal is generated based on the excitation chirp and subsequently, for each pixel of the image, the synthesized reference chirp is cross-correlated with the photothermal relaxation signal of that pixel, recorded by the mid-IR camera. The cross-correlation (CC) evolves as the thermophotonic waves generated inside the sample reach the interrogated surface conductively, radiatively or in combination^26,27^. These thermal transients carry information about the morphology and optical and thermal properties of the layers beneath the surface, with information from deeper layers taking a longer time to arrive at the surface^34^. The CC is calculated at time intervals determined by “slice width”, *W*_*T*_, and an “incremental delay unit”, *d*, where *W*_*T*_ is controlled by the operator and is used for the calculation of *d* (Fig. 2). Next, the calculated CC is truncated by a time gating filter to provide depth resolved information. Finally, for each *d*, compilation of the data from all pixels leads to a depth-resolved 2D “slice” image of the sample, and the compilation of 2D slices results in the 3D model of the sample. In this manner, for each pixel, three CC contrast channels were generated and used for the current study: amplitude peak, phase and zero-phase delay time^26^. In eTC-PCT, the amplitude channel correlates with the intensity of the thermal relaxation signal, while the phase-based channels correlate with the difference in arrival time of the thermal signal at surface from different points inside the sample. The zero-phase delay channel is a post-processing of the phase output, correlating the phase information with zero net thermal flux in the material, thus highlighting the regions with highest thermal signal generation (such as material-air boundaries) under conditions equivalent to insulating boundaries.

Importantly, eTC-PCT axial resolution is determined by both the sample composition and *W*_*T*_. A longer *W*_*T*_, as used in this study, will compile data from more recorded frames for CC calculation of each pixel at each delay time and thus results in higher contrast and SNR, at the cost of reduced axial resolution.

As the diameter of the incident laser beam in our setup is considerably larger than the thermal diffusion length within the material, the thermal-wave problem can be considered to be one dimensional. In this case, for a turbid medium such as a tooth, the pulse-chirp photothermal relaxation signal used for eTC-PCT can be derived as^35^:

1 $$S(t)=C\frac{{\mu }_{a}{\mu }_{IR}}{\rho c({{\mu }_{IR}}^{2}-{{\mu }_{eff}}^{2})}(\begin{array}{c}A[\begin{array}{c}\,{\mu }_{IR}f({{\mu }_{eff}}^{2}\alpha t)+\\ {\mu }_{eff}f({{\mu }_{IR}}^{2}\alpha t)\end{array}]\\ +B[\begin{array}{c}\,{\mu }_{IR}f({{\mu }_{tr}}^{2}\alpha t)+\\ {\mu }_{tr}f({{\mu }_{IR}}^{2}\alpha t)\end{array}]\end{array})\ast Exc$$

with

$$Exc=\mathop{\sum }\limits_{m=0}^{p}\,\delta [t-(\frac{-{\omega }_{1}+\sqrt{{\omega }_{1}^{2}+2\pi r(4m+1)}}{2r})]$$

where, *ρc* is the volumetric specific heat of the sample, *t* is time, *μ*_*a*_ is the medium’s optical absorption coefficient at the excitation wavelength, *α* is the thermal diffusivity and $f(x)={e}^{x}erfc(\sqrt{x})$ with *erfc* signifying the complementary error function. *μ*_*tr*_*, μ*_*IR*_ and *μ*_*eff*_ are the transport coefficient, IR absorption coefficient and effective attenuation coefficient $\sqrt{3{\mu }_{tr}{\mu }_{a}}$, respectively. Constants *A*, *B* and *C* are defined in ref.^25^. In this study, the excitation signal (*Exc*) is a linear frequency modulated (LFM) pulsed waveform where *ω*_1_and *ω*_2_ are the starting and final angular frequencies, *r* = (*ω*_2_ − *ω*_1_)/*T* is the sweep rate, and *T* is the period of the LFM chirp. *m* = *0, 1, 2, … p*, where *p* + *1* is the number of pulses to be generated. *δ* is the Dirac delta function.

### Tooth imaging *In Vitro*

**Figure 3:**
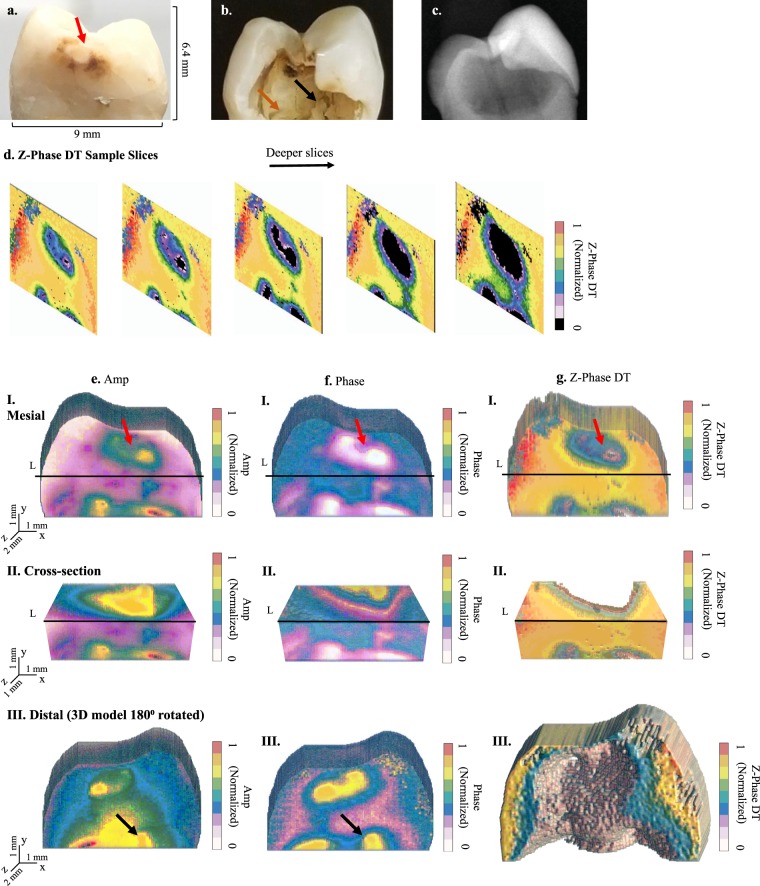
eTC-PCT 3D reconstruction of advanced decay. (**a**–**c**) Show the mesial and distal surfaces of sample 1 along with its x-ray image, respectively. Only the mesial face was interrogated. Sample 1 features a large, cavity-like, carious lesion, only visible from the distal surface, with an inner wall-thickness of 1.6 mm behind the mesial surface. (**d**) Shows a few sample tomographic eTC-PCT slices of zero-phase delay data for this tooth. Tomographic cross-correlation slice image evolution from the mesial surface inward is shown from left to right as indicated by the horizontal arrow. (**e**–**g**) 3D reconstruction of sample 1 using eTC-PCT tomographic slices from amplitude, phase, and zero-phase delay time channel data, respectively. For all channels: row I shows the mesial face. Row II is a transverse cross-section of the reconstructed 3D model, cut along line L, as marked in row I images. The extent of the lesion inside the tooth is seen in the cross-section. Note that the z-axis scaling is different in this row for better visualization. Row III is the 180 degree rotation of the 3D model in the software, showing the sample’s reconstructed distal face. The data clearly demonstrate the tomographic 3D capabilities of eTC-PCT. The asymmetric boundaries of the lesion, seen in visible light, are reconstructed in the 3D model. The zero-phase-delay-time channel column (**g**) provides the best structural modeling and boundary delineation capability due to its unique property of sharply imaging the zero-net-thermal-flux instant/depth for each pixel. Color bars have arbitrary units.

**Figure 4:**
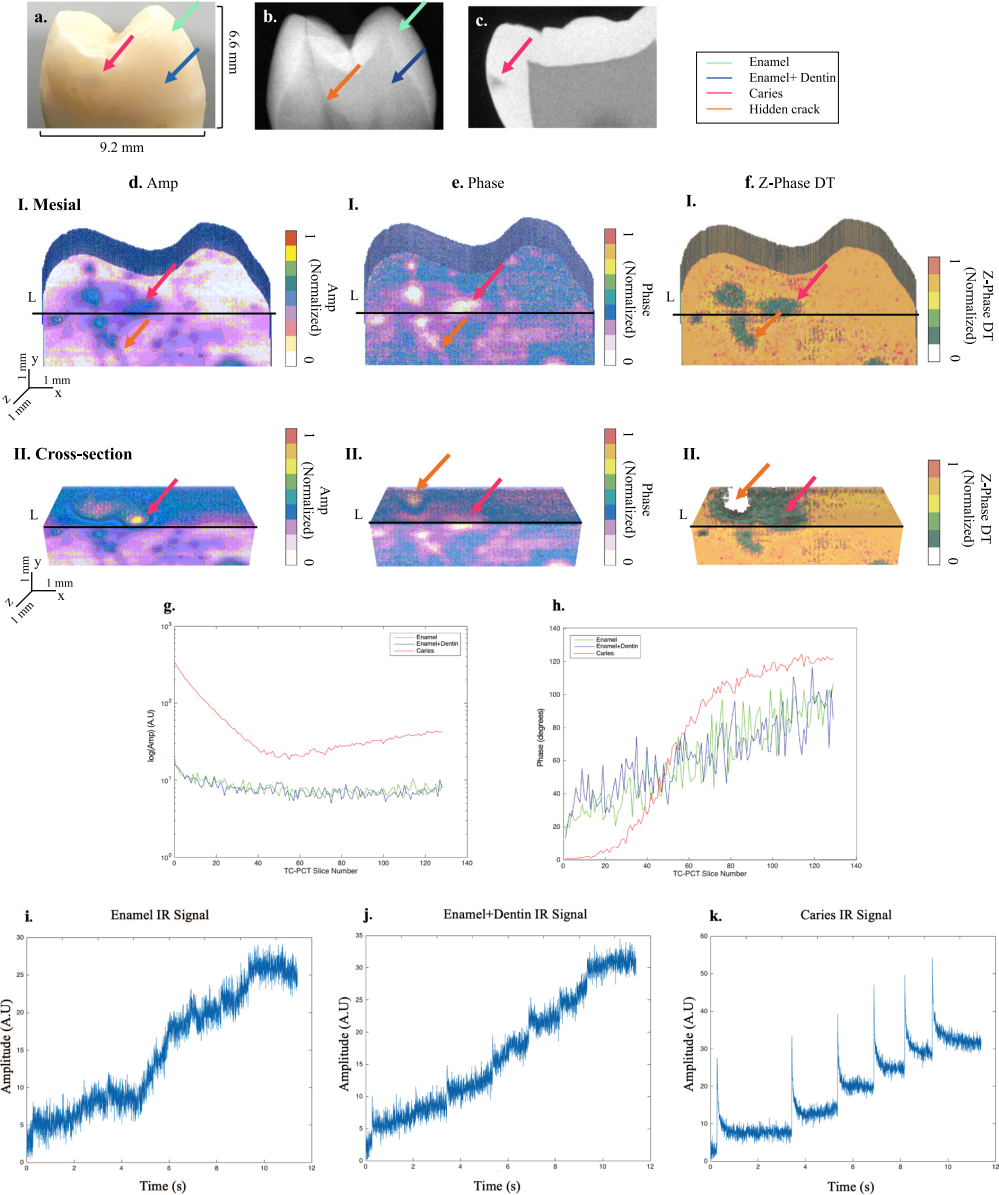
Application of eTC-PCT to early caries. (**a**,**b**) Show the interrogated mesial surface of sample 2 and its x-ray image, taken perpendicular to the carious surface. (**c**) Is a sagittal micro-CT slice image, showing the location and extent of the caries. In total, 4 regions of interest are marked in (**a–c**) by color arrows: initial caries (not seen in x-ray), enamel, and enamel-covered dentin, and hidden crack (only seen in x-ray). (**d–f**) Present the 3D reconstruction based on eTC-PCT amplitude, phase and zero-phase delay time channel data, respectively. The imaging direction relative to the carious surface is similar to x-ray. The carious region is distinctly visible in all three channels, with the amplitude channel exhibiting finer detail compared to the phase-based channels. The hidden-crack is visible in the amplitude and phase channel outputs. For all channels: row I shows the interrogated mesial face. Row II is a transverse cross-section of the reconstructed 3D model, cut along line L, as marked in row I images. Caries is seen extending inside the tooth over the cross-section. (**g,h**) Present the plotted eTC-PCT amplitude and phase data for healthy enamel, healthy enamel-covered dentin and caries. The carious region exhibits the most distinct signal with the highest initial amplitude and lowest initial phase lag (up to slice number ~45, before rising and saturating beyond slice number ~65), with minimal noise compared to the other two regions. (**i**–**k**) Show examples of the raw IR thermal relaxation signal from each region, with caries having the highest signal-to-noise ratio due to significantly higher photon absorption. The enamel-covered dentin exhibits less noise in its relaxation signal, compared to the pure enamel region, due to the presence of the higher-absorption-coefficient dentinal underlayer. This sample demonstrates the eTC-PCT capability for high-contrast depth profiling of early dental caries perpendicular to imaging direction, when x-rays are insensitive in the same scenario. Color bars have arbitrary units.

**Figure 5:**
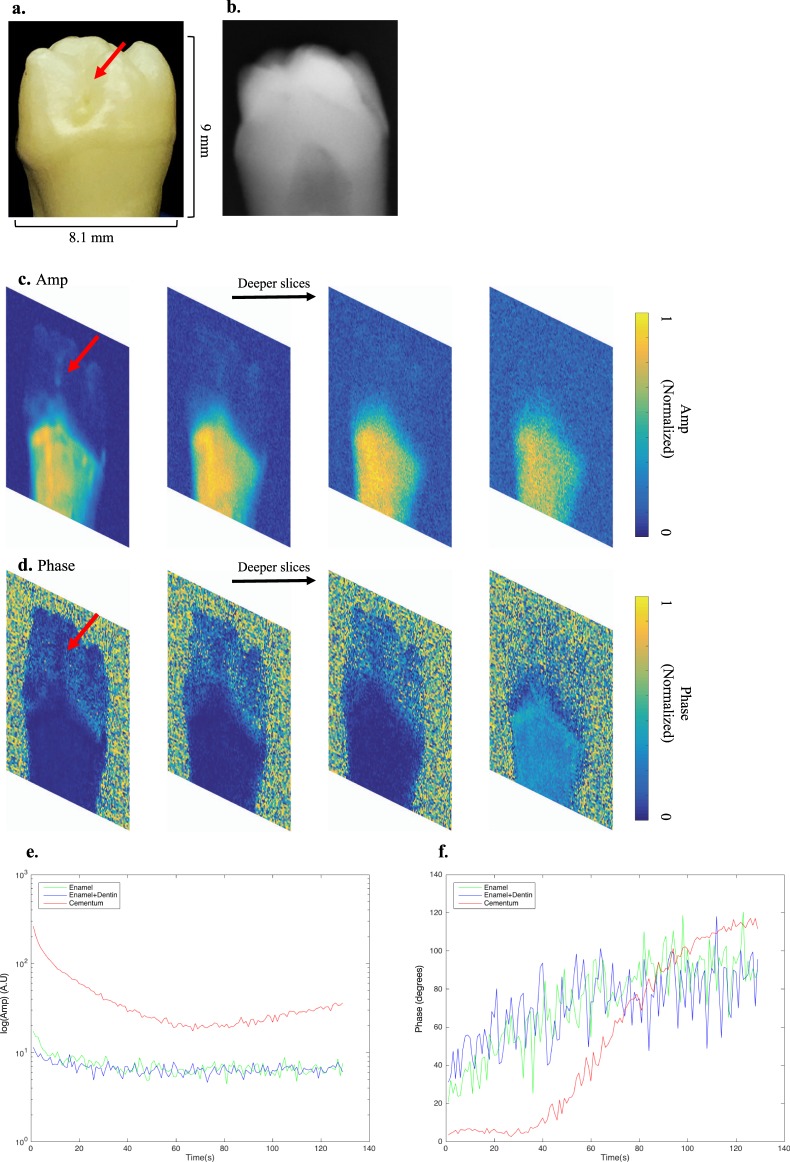
eTC-PCT results for healthy teeth. (**a,b**) Show the interrogated surface of tooth sample 3 and its x-ray image. This sample is healthy, as confirmed by a clinical dentist’s visual inspection, x-rays and the Canary system. (**c,d**) Show sample slices of eTC-PCT tomographic reconstructions of amplitude and phase data for this sample. Tomographic cross-correlation slice image evolution from the surface inward is shown from left to right as indicated by the horizontal arrow. The tooth cementum is seen with high contrast due to its high absorption at 808-nm. On the tooth crown, other than a natural surface pit (red arrow) seen in the initial cross-correlation slices, no other discernible feature is observed in the eTC-PCT image. This is due to the relative transparency of healthy enamel to the 808 nm wavelength. (**e**,**f**) Show the plotted eTC-PCT amplitude and phase data for enamel, enamel-covered dentin and cementum. The regions on the healthy tooth crown exhibit low amplitude and highly noised phase data with larger lag. The highly absorbing cementum exhibits significantly higher amplitude and notably less noisy phase data with smaller initial phase lag.

**Figure 6:**
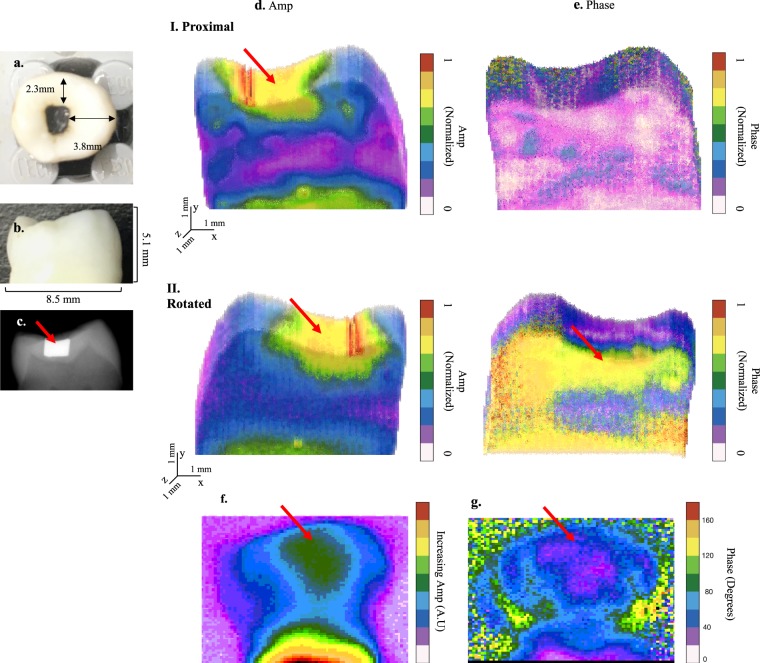
eTC-PCT application to teeth with amalgam filling. Occlusal (**a**) and proximal (**b**) surfaces, and the x-ray image (**c)** of sample 4. This sample contains an amalgam filling (red arrow), visible only from the occlusal surface, at a distance 2.3 mm behind the proximal surface and 3.8 mm behind the lingual surface. (**d**) and (**e**) 3D reconstruction of the sample based on eTC-PCT amplitude and phase data, respectively, for imaging the proximal surface. Row I shows the interrogated proximal surface. In row II the model is rotated by 180 degrees. Due to the contribution of both radiative and conductive heat transfer information to the amplitude channel, the highly absorbing amalgam is visible in all amplitude slices. The phase data, however, are solely based on conductive, time-dependent, information and thus the filling becomes visible in this channel in the last slices. The bright region at the bottom is the tooth cementum, due to its high absorption coefficient at our excitation wavelength. (**f**) and (**g**) show one slice (slice width = 60 ms) from each of the amplitude and phase channels of eTC-PCT imaging results for the lingual surface of sample 4. The filling is located ~3.8 mm below that surface and is seen in the eTC-PCT output (indicated by the red arrow). This result demonstrates both eTC-PCT’s ability in detecting this material as well as its penetration depth range. Color bars have arbitrary units, unless specified.

Four extracted human molar teeth with different conditions, each of various degrees of health, were used for this study, shown in Figs 3(a,b), 4(a), 5(a) and 6(a,b). Visual assessment and ranking was done using ICDAS II (“International Caries Detection and Assessment System”) Criteria^36^. Two dentists, trained in using the ICDAS II visual scoring system, independently scored each tooth surface. Tooth sample 1 had a large carious lesion, manifested as a “hole” or “cavity”, visible only from the distal surface. The deepest point of the lesion was measured, using a caliper, to have a “wall-thickness” of ~1.6 mm below the mesial enamel surface which is the imaged surface. The mesial and distal surfaces were given ICDAS II scores of 3 and 6, respectively. Tooth sample 2 had faint traces of caries on its mesial face, as seen in visible light. This sample was given an ICDAS II score of 1. Moreover, using micro-CT (μCT) as the non-destructive gold standard, the sample was determined to have initial caries in the outer half of its enamel which corresponds with its ICDAS II classification^37^. The presence of early caries was also confirmed using the PTR-LUM Canary System. Tooth sample 3 was healthy, as confirmed through visual ranking of 0 using the ICADS II system, an x-ray, and the Canary System. Tooth sample 4 had an amalgam restoration, only visible from the occlusal surface of the tooth, 2.3-mm distance below the mesial surface and 3.8 mm below the lingual surface. This sample was given an ICDAS II score of 0. The samples were chosen to explore the performance capabilities of eTC-PCT for different dental scenarios. In all cases, the investigated lesions were either located on the imaged surface (early caries on sample 2), or visually hidden behind the imaged surface (samples 1 and 4).

As detailed in the methods section, for each sample, the laser chirp frequency sweep range and duration (from 12 s for healthy teeth and early caries to 80 s for highly detailed 3D reconstruction), the excitation pulse width (ranging between 20 ms and 80 ms) and the reconstruction *W*_*T*_ (ranging between 10 ms and 60 ms), were chosen for an optimal combination of image contrast and axial resolution. In general, lower frequencies (resulting in a longer thermal diffusion length), longer excitation pulse width (larger amounts of deposited energy) and longer chirp duration (allowing ample time for the arrival of information to the surface) were employed to evaluate depth penetration and high-accuracy structural characterization capabilities of eTC-PCT in teeth. In contrast, higher chirp frequencies and shorter chirp durations are sufficient for imaging near-surface lesions, such as early caries.

Figure 3 presents the eTC-PCT imaging results for the mesial surface of sample 1 and also reveals the subsurface structure, hidden behind the imaged surface, upon rotation of the tomographic 3D reconstruction of the tooth. The complex structure of this sample, as seen in visible-light images (Fig. 3(a,b)), provides an opportunity for clear demonstration of the tomographic nature of the eTC-PCT data and its 3D reconstruction capabilities. To this end, Fig. 3(d) presents a number of representative eTC-PCT CC slices for sample 1. These slices are reconstructed using the zero-phase delay channel data and demonstrate the evolution of the system output based on thermal data from increasingly deeper subsurface points. All eTC-PCT channels provide tomographic sliced data, which can then be complied to reconstruct a 3D model. In both amplitude and phase channel 3D reconstructions, Fig. 3(e.I–f.III), the boundaries of the lesion can be observed in a transverse plane across the tooth at virtual cross-section designated by the line L. Furthermore, the visible asymmetric outlines of the cavity-like lesion on the distal surface, Fig. 3(b), have also been captured by both channels, as seen in the 180° rotated Fig. 3(e.III) and 3 (f.III). Red and black arrows mark two representative, distinguishable points of interest which are seen both in visible light images and in the eTC-PCT reconstructions. The zero-phase delay time channel data provide the most visually accurate structural characterization of the tooth subsurface, with unprecedented true 3D reconstruction of the lesion structure, including the sharpest definition of the various hard-dental-tissue boundaries to-date, as seen in Fig. 3(g.I–g.III).

The high contrast between the intact tooth tissue and the lesion in amplitude and phase channels stems from two superposed and mutually amplifying factors: a) The higher than intact enamel absorption coefficient within the lesion boundaries at our chosen excitation wavelength resulting in higher amplitude and smaller phase lag; and b) the highly reduced thermal conductivity of the lesion. They both contribute to increased contrast in the photothermal signal. In the zero-phase delay time channel, the thermal-wave energy confinement in the absorption region delineates the boundary at which the net thermal flux becomes zero, resulting in the sharp imaging of boundary morphologies as in Fig. 3(g.III), unlike the diffusive boundaries witnessed in conventional thermographic images.

The imaging results for the mesial surface of sample 2 with enamel caries are presented in Fig. 4. Visible-light, eTC-PCT and X-ray imaging were done on the coronal plane of the tooth (i.e. imaging direction perpendicular to carious surface). In the visible-light image of the sample’s mesial surface, Fig. 4(a), colored arrows mark samples from each of the early caries (red), enamel (green), and enamel-covered dentin (blue). A hidden crack is visible in the x-ray image (Fig. 4(b)) and is marked with an orange arrow. The early caries is not seen in the x-ray image. The results from the μCT scan are presented in Fig. 4(c), where the proximal caries is visible in a sagittal image of the tooth. μCT confirms the presence of initial caries in the outer half of the enamel (red arrow). Figure 4(d.I–f.II) show the eTC-PCT reconstructions of this sample in the amplitude, phase and zero-phase delay time channels, respectively. The carious region is clearly seen in all channels due to a high degree of photon absorption. The shallow depth of the enamel caries seen in these images corresponds to μCT results, confirming the profilometric capability of eTC-PCT in caries visualization. The fine detail of the hidden crack is best visible in the amplitude and phase channels. The zero-phase delay time channel is clearly correlated with the amplitude and phase channels within highly absorbing regions, reaching close to zero in caries (red arrow) or zero in the hidden crack (seen as empty space marked with orange arrow).

The slice-by-slice evolution of the outputs from amplitude and phase channels is plotted in Fig. 4(g,h). It is observed that the phase lag increases with the slice number, as the depth increases. Conversely, we expect the amplitude to be at its peak at the surface image and to drop with increasing depth into the sample. The carious region exhibits a much higher initial amplitude signal, considerably smaller initial phase lag before eventual rise due to increasing depth and subsequent saturation, and the least noise compared to other regions. This is a direct result of the significant light absorption in carious regions at our selected wavelength of 808 nm. The enamel and enamel-covered dentin regions are comparatively transparent to this wavelength, resulting in marginal photon absorption and thermal wave generation. This translates into considerably smaller eTC-PCT amplitude and highly noised phase data with larger initial lag. The raw IR signals from 3 individual pixels, each belonging to one of the aforementioned regions and recorded by the IR camera, are shown in Fig. 4(i–k). In the enamel-covered dentin area, the IR signal, Fig. 4(j), has higher SNR compared to the IR signal of the pure enamel region, Fig. 4(i), because of the smaller thermal diffusivity of dentin compared to enamel that induces shallow confinement^38,39^.

Sample 3 demonstrates eTC-PCT imaging of a healthy tooth. The imaging frame for this sample was set to include both the crown and root of the tooth, Fig. 5(a). 3D reconstruction for this sample was hard to achieve, since, as mentioned above, healthy enamel and enamel-covered dentin are relatively transparent to the 808 nm wavelength, providing low contrast amplitude and noised phase data with large lag. The cementum, on the other hand, is highly absorbing at this wavelength, resulting in higher initial amplitude data and smaller phase lag. eTC-PCT sample output slices using amplitude and phase channels are presented in Fig. 5(c,d), respectively. A natural surface depression (pit) marked by a red arrow on the interrogated face, is also observed in shallow (early-delay-time) amplitude and phase images. These data are also plotted slice-by-slice, for all slices, in Fig. 5(e,f). In this case, zero-phase delay time provides no contrast data from non-absorbing scattering media (i.e. intact enamel) capable of being used for image reconstruction.

Sample 4, Fig. 6(a,b), is used for evaluating the penetration depth ability of dental eTC-PCT by comparing the output images with the location of the amalgam filling as measured in visible light. Shown here is one of the interrogated sides of sample 4 (proximal surface) as well as the amalgam filling, the latter being visible solely from the occlusal surface. Figure 6(d,e) show the 3D reconstruction of the eTC-PCT amplitude and phase data for this sample, where the filling is marked with a red arrow. This reconstruction has been created with the imaging data from the proximal face, with the filling at depth of ~2.3 mm below this surface. Notably, while the filling is clearly seen only in the last few slices of the phase data, it is visible practically in all amplitude slices, despite its depth below the interrogated surface. This observation will be explored in the discussion section below and is interpreted in conjunction with Fig. 6.

The filling was also detected from the sample’s lingual face at an even higher penetration depth of ~3.8 mm, Fig. 6(f,g). However, this case proved unsuitable for 3D reconstruction. The considerably higher energy deposition due to the longer pulse duration used for deep penetration (80 ms), as well as the use of a longer slice width (60 ms) to enhance the contrast of the signal from deeper into the sample, significantly reduced the depth-resolved properties and axial resolution of the eTC-PCT images, both requirements for correct 3D reconstruction.

## Discussion

eTC-PCT produces imaging outputs based on both the photothermal relaxation signal amplitude and phase data. In general, following light absorption in a scattering medium, in addition to the conductive heat transfer information generated inside the sample and reaching the interrogated surface, direct infrared (thermal radiation) photons are also emitted from all absorption sites. In human teeth, a fraction of these thermal photons pass through unhindered^40^ and contribute to the prompt IR signal recorded by the camera. The thermophotonic photothermal images thus generated from inside a scattering medium carry optical as well as thermal information from thermal diffusion depths determined by the laser modulation frequency (conductive)^41^ and/or longer depths (radiative).

Crucially, while conductive thermal waves reach the interrogated surface with phase values indicative of the depth and physical characteristics of their generation site, the aforementioned direct IR radiated emissions travelling at the speed of light reach the surface from all depths virtually at the same time, with no measurable phase difference relative to each other. As such, the eTC-PCT phase channel contains conductive heat transfer information, which is time (and thus depth) dependent. The practical implication of this in eTC-PCT is that the phase and zero-phase delay time channels output data provide comparatively more accurate representations of absorber depths, and thus, in general, are more suitable than amplitude images for constructing depth-resolved, tomographic images of teeth. The amplitude channel data, however, are a superposition of both conductive thermal waves and direct co-modulated infrared radiation. Therefore, amplitude signals are more suitable for obtaining the highest contrast and detail (particularly compared to the zero-phase delay time channel), but are less accurate in providing information about the relative depth of the absorbers. These differences are clearly seen in the case of the amplitude and phase reconstructions of sample 4 and the generally finer radiative resolution observed in the amplitudes of all samples. It must be noted that both phase and amplitude channels provide useful depth information up to a saturation point, as seen in the plots for samples 2 and 3, Figs 4 and 5, respectively.

The depth profilometry capability of eTC-PCT was exploited in the dental results presented in this study for the reconstruction of a large cavity-like carious lesion and the detection of an amalgam filling in samples 1 and 4, respectively. The high sensitivity of eTC-PCT to dental caries was demonstrated for sample 2. For this sample, while the marked carious lesion is barely seen in visible light, the area is prominently present in the eTC-PCT phase based images and the subsurface carious extent is revealed in both amplitude and phase channels. This outcome is based on the fact that demineralized regions cause light absorption which results in highly localized optical-to-thermal-wave conversion from these regions with energy contained within a truncated CC time-delay slice. As such, after each laser pulse, photothermal energy confinement within the demineralized or otherwise carious region results in a higher SNR and a slower decay rate for the photothermal transient compared to healthy thermally non-confined regions.

Notably, the comparison of sample 2 results with those from sample 3 reveal a general mechanism of how early enamel caries (confirmed by micro-CT and the Canary System) manifests itself in eTC-PCT images. In both samples, healthy enamel is identified by low absorption, highly noised phase and essentially no contrast data in the zero-phase delay time image which acts as an *ad hoc* criterion for the health status of a tooth.

Sample 2 results also mark a significant advantage for eTC-PCT over traditional dental X-rays. Unlike X-rays, eTC-PCT is capable of high-contrast visualization of early enamel caries on an interrogated region perpendicular to the beam direction. This capability will be further explored in future studies for the case of early occlusal caries, another common x-ray shortcoming. It must also be noted that, since thermophotonics-based technologies work on the basis of photon absorption and thermal wave generation, other absorbing regions on a tooth, such as the cementum or a crack, also exhibit strong photothermal signals with similarities to caries. As a result, currently eTC-PCT highlights the defective regions as regions of interest to be further investigated. In this process, the information from the different channels of eTC-PCT, its depth profilometry capability, and the visible structural features of the tooth can be helpful tools in identifying the signal origin. As an example, for sample 2, while the hidden crack looks similar to caries in the surface layer of the eTC-PCT channels, it is distinguishable from the caries in the subsurface layers in both phase (appearing deeper than the near-surface caries) and zero-phase delay channels (appearing as an empty space).

In summary, we have reported the first dental three-dimensional eTC-PCT images featuring operator controlled axial resolution, a lateral resolution of 33 µm, depth profilometric capability and penetration depth of up to ~ 3.8 mm, depending on the investigated defect’s structural and thermal properties. Case studies of the ability of this modality to provide 3D images of early enamel caries, advanced decay and amalgam restorations were presented, indicative of potential multifaceted clinical applications of dental eTC-PCT. Being a safe non-ionizing and maximum-permissible exposure (MPE) compatible system, eTC-PCT is suitable for the detection and frequent monitoring of dental caries and the over-time response of lesions to treatment. Accordingly, based on the successful outcome of the current work, future studies will focus on imaging and longitudinal assessment of controlled artificial early carious lesions on varying locations (e.g. buccal, proximal, occlusal), in order to establish the eTC-PCT caries detection threshold and its sensitivity for monitoring lesion progression in different scenarios.

## Materials and Methods

### eTC-PCT system specifications

An IR camera (A6700sc, FLIR, USA, 3–5 μm spectral response), recorded the thermal evolution of the sample following exposure to laser irradiation. A linear frequency modulation (LFM) chirp was produced using a function generator (Keysight 33500B, USA). The LFM chirp signal, the excitation chirp, controlled the diode laser (Jenoptic JOLD- 120-QPXF-2P) through a laser driver (PCO-6131, Directed Energy, Colorado, USA), and was recorded using a high-speed data acquisition module (NI PCI-6281) for synthesizing the reference chirp. The laser beam was passed through a collimator (F22SMA-B, Thorlabs Inc., New Jersey, USA), and a diffuser (ED1-C20, Thorlabs Inc., New Jersey, USA) to become expanded, homogenized and collimated. A camera frame rate of 104 Hz and a frame size of 8.5 mm by 10.5 mm was employed in the dental studies. For an image size of 320 × 256 pixels, a lateral resolution of approximately 33 μm was calculated. The axial resolution is dependent on sample composition and the selection of the *W*_*T*_ parameter (discussed below in “Imaging and reconstruction Parameters” section**)**. The Enhanced TC-PCT reconstruction algorithm was utilized to create 2D depth planar and 3D temporally truncated cross-correlation (CC) images of tooth samples.

### Sample collection and storage

Following the research ethics guidelines at University of Toronto, anonymous extracted human teeth were collected from University of Toronto dentistry department. No cleaning or modifications other than the removal of soft tissues were performed on the teeth. Each tooth was mounted on a Lego brick using epoxy to provide a stable platform for imaging. The teeth were kept refrigerated and stored in distilled water, to keep them hydrated and at a constant humidity level.

### Safety

All experiments, including sample preparation, were conducted in accordance with bio- and laser-safety regulations of the University of Toronto.

### Imaging and reconstruction parameters

Imaging parameter optimization and determination for each dental scenario was accomplished on the basis of MPE compatibility, varying desired inspection depth for each sample, the thermophotonics principles, and experimentation. The mesial surface of sample 1 was imaged using a laser chirp sweep range of 0.03–0.1 Hz, duration of 80 s and a pulse width of 40 ms, and reconstructed with *W*_*T*_ = 40 ms. The proximal surfaces of samples 2 and 3 were imaged using a laser chirp sweep range of 0.2–0.6 Hz, duration of 12 s and a pulse width of 20 ms, and reconstructed with *W*_*T*_ = 10 ms. The proximal surface of sample 4 was imaged using a chirp sweep range of 0.04–0.3 Hz, duration of 35 s, and pulse width of 20 ms, and was reconstructed with *W*_*T*_ = 40 ms. This sample’s lingual surface was subject to the same settings as sample 1, with the exception of pulse width increase to 80 ms, and *W*_*T*_ increase to 60 ms. A longer *W*_*T*_, used for deeper interrogation scenarios, enhances the contrast at the cost of decreased axial resolution. For each sample, *W*_*T*_ was chosen to provide the optimal contrast/resolution combination. The highest axial resolution, at 63 μm, was measured for *W*_*T*_ = 10 ms.

### 3D data reconstruction

The slice-by-slice eTC-PCT cross-correlation data for the amplitude, phase and zero-phase-delay-time channels was saved in LabView for all camera pixels. For phase and amplitude channels, time slices were only used up to the saturation point of a subsurface absorber inside the medium (e.g. caries for the 808 nm wavelength). As an example, the phase channel for sample 2 became saturated after approximately 65 slices, Fig. 4(g). For the zero-phase delay time channel, the same number of slices was used as with the amplitude channel. After extracting the pre-saturation slices, these data were either directly used for 3D reconstruction through ImageJ software^42^ or imported into MATLAB for data analysis of the three channels. Using ImageJ for each channel, the slice images are imported as a stack and compiled in 3D using the Volume Viewer plugin. These images are not on the same scale, and the software does not allow for associating quantitative values to image colors in the volume viewing format. For samples 1 and 2, trilinear interpolation with 2.5 sampling rate was used.

### Micro-computed tomography

CT imaging of the tooth sample was acquired using a Phoenix X-ray V|tome|x 240 kV μCT system (General Electric Sensing and Inspection Technologies), with a voxel resolution of 24 μm. Image reconstruction was performed in Phoenix X-ray Datos|x-reconstruction software and further image processing was performed in ImageJ software.

### Photography

All photographs were taken using a 12MP digital camera with a 29 mm, f/2.2 lens.

### Pulp chamber temperature measurement

A T-type thermocouple was connected to a temperature measurement unit (OMEGA, CNi32 series) and inserted into the pulp chamber from the occlusal surface of the tooth, while the buccal surface was subject to laser excitation during eTC-PCT imaging.

## Data Availability

Data supporting the results reported in this work can be obtained by contacting the corresponding author.
